# C/EBPβ Participates in Nerve Trauma-Induced TLR7 Upregulation in Primary Sensory Neurons

**DOI:** 10.1007/s12035-022-02763-0

**Published:** 2022-02-09

**Authors:** Long He, Jing Cao, Bao-Chun Jiang, Jian-Jun Yang, Yuan-Xiang Tao, Yanqiu Ai

**Affiliations:** 1 Department of Anesthesiology, Pain and Perioperative Medicine, The First Affiliated Hospital of Zhengzhou University, Zhengzhou, 450052 Henan China; 2 Department of Anatomy, College of Basic Medicine, Zhengzhou University, Zhengzhou, 450001 Henan China; 3 Institute of Pain Medicine and Special Environmental Medicine, Nantong University, Nantong, 226019 Jiangsu China; 4 Department of Anesthesiology, New Jersey Medical School, Rutgers, The State University of New Jersey, Newark, NJ 07103 USA

**Keywords:** TLR7, C/EBPβ, Dorsal root ganglion, Nerve trauma, Neuropathic pain

## Abstract

Nerve trauma-induced toll-like receptor 7 (TLR7) expression level increases in primary sensory neurons in injured dorsal root ganglion (DRG) avails to neuropathic pain, but the reason is still unknown. In the current study, we showed that unilateral lumbar 4 (L4) spinal nerve ligation (SNL) upregulated CCAAT/enhancer-binding protein-β (C/EBPβ) expression in ipsilateral L4 DRG. Preventing this elevation attenuated the SNL-induced upregulation of TLR7 in the ipsilateral L4 DRG and inhibited cold/thermal hyperalgesia and mechanical allodynia. In injected DRG, mimicking nerve trauma-induced C/EBPβ upregulation increased TLR7 levels, augmented responses to cold/thermal/mechanical stimuli, and caused ipsilateral spontaneous pain with no SNL. Mechanistically, SNL upregulated binding of increased C/EBPβ to *Tlr7* promoter in ipsilateral L4 DRG. Accorded that C/EBPβ could trigger the activation of *Tlr7* promoter and co-expressed with *Tlr7* mRNA in individual DRG neurons, our findings strongly suggest the role of C/EBPβ in nerve trauma-mediated TLR7 upregulation in injured primary sensory neurons.

## Introduction

Neuropathic pain refers to chronic pain resulting from central or peripheral nervous system (CNS, PNS) disease or injury, which poses a massive challenge to physicians due to poor response to conventional pain treatments. About 3.3–17.9% of the USA and European population suffers from neuropathic pain [1]. It is identified based on persistent pain of spontaneous onset, cold/thermal hyperalgesia, spasmodic burning pain, and allodynia. Currently, some treatments are available to treat neuropathic pain, like non-steroidal anti-inflammatory drugs (NSAIDs), opioids, antidepressants, and anti-convulsants [1–3]. Nonetheless, many cases do not achieve sufficient pain relief using the existing therapeutics since they mostly lack specificity to neuropathic pain [1, 3]. Thus, discerning the underlying mechanisms of neuropathic pain could provide new and more efficient therapeutic strategies for treating neuropathic pain.

Hyperexcitability and abnormal deranged firing in primary sensory neurons in the dorsal root ganglion (DRG), as well as neuroma in the injury position, have been proposed to promote neuropathic pain in the PNS following peripheral nerve injury [2, 4]. DRG neurons’ aberrant spontaneous activity together with consequently heightened neurotransmitter release from their primary afferent may be associated with maladaptive changes, just as enzyme and receptor translation, gene transcription, together with voltage-and ligand-dependent ion channels in DRG [5–8]. In mammals, Toll-like receptors (TLRs) play essential roles in inducing the innate immune responses to molecular patterns related to a pathogen. Previous findings confirm that the TLRs family contains 13 proteins (TLR1–13) in mammals. Toll-like receptors 7 (TLR7) recognize single-stranded nucleic acids from RNA viruses and signals via myeloid differentiation factor 88 (MyD88) the production of cytokines and chemokines to fight against pathogenic infection [9].

Cumulative evidence suggests that TLR7 is highly implicated in abnormal pain hypersensitivity and itch. Approximately 33% of DRG neurons are positive for TLR7, about 55% are small neurons [10]. TLR7 is co-expressed with TRPV1, GRP, TRPA1, and MrgprA3 in DRG neurons [11, 12]. Natural and synthetic TLR7 ligands are potent adjuvant for rapid action potential and inward current of nociceptive neurons in DRG [11]. In our previous study, trauma to the PNS resulting from spinal nerve ligation (SNL) significantly increased TLR7 expression in injured DRG neurons at mRNA and protein levels [10]. Blocking this increase by virus-mediated knockdown of TLR7 in DRG could alleviate the cold-/thermal-related nociceptive hypersensitiveness and SNL-mediated mechanical allodynia in mice, regardless of gender. Mimicking this increase by overexpression of TLR7 in naïve mice DRG could elicit neuropathic pain symptoms, including hypersensitivity to mechanical, heat, and cold. Mechanistically, elevated TLR7 promoted the p65 (NF-κB transcription factor (TF) family members) nuclear import and phosphorylation in the injured DRG neurons [10]. In primary sensory neurons, peripheral nerve trauma-induced DRG TLR7 increase mainly induced neuropathic pain through the activation of the NF-κB pathway [10]. In addition, in the surgical osteoarthritis (OA) rat model, the upregulated TLR7 in synovial tissue mediated knee OA pain, as single intra-articular injection of TLR7–9 antagonist exerted long-term analgesia [13]. Nevertheless, its role in chronic pain conditions is still elusive.

CCAAT/enhancer-binding protein-β (C/EBPβ) belongs to the transcription factor family, which has a high conservation degree of the DNA binding-related basic-leucine zipper domain [14]. During the development of inflammatory hypersensitivity, C/EBPβ-Runx1-containing transcription regulatory complexes recruited the TRPV1 gene promoter to modulate TRPV1 expression in DRG neurons [15]. In the HIV gp120-induced neuropathic pain state, phosphorylated C/EBPβ triggered by TNFα/TNFRI-pCREB signaling pathway contributed to HIV-related neuropathic pain [16]. Furthermore, our previous study showed that peripheral nerve trauma caused by chronic constriction injury (CCI) of the sciatic nerve increased the expression of C/EBPβ in the injured DRG and that this increase contributed to the CCI-induced neuropathic pain induction and maintenance [17]. The evidence indicates the involvement of DRG C/EBPβ in neuropathic pain genesis.

We adopted the JASPAR database and identified the consensus-binding motifs (− 138 to –128 and –56 to –46) of C/EBPβ in the *Tlr7* gene promoter, strongly suggesting that C/EBPβ likely participated in the SNL-triggered transcriptional activation of the *Tlr7* gene. Given that nociceptive hypersensitivity is caused by direct nerve injury in the SNL model [18, 19] but initiated primarily by ischemia in the CCI model [20], we first examined whether SNL, like CCI, increased the level of C/EBPβ expression in injured DRG in the present study. We also examined whether blocking the increased DRG C/EBPβ alleviated SNL-induced elevation of TLR7 in injured DRG and nociceptive hypersensitivity. The effects of mimicking SNL-induced DRG C/EBPβ increase on DRG TLR7 expression and basic nociceptive threshold were observed. Finally, whether C/EBPβ directly bonded to and triggered the *Tlr7* promoter in injured DRG after SNL was investigated.

## Materials and Methods

### Animal Preparation

The present work adopted 7–8-week-old CD-1 male adult mice provided by Charles River Laboratories (Beijing, China). All mice were raised within a housing room under 12-h/12-h light/dark cycle conditions and were allowed to eat food and drink water freely. Our study protocols were approved by the Institutional Animal Care and Use Committee at Zhengzhou University, China. The animals were adaptively fed for 2–3 days prior to behavioral tests. The treated groups were blinded for experimenters.

### Neuropathic Pain Models

In accordance with the previous description, we created a mouse model of neuropathic pain using lumbar 4 (L4) SNL [21–24]. Briefly, under 1.5–2.5% sevoflurane inhaled anesthesia and on a warm surface, an incision was made in the midline of lower back skin on L5–L2 vertebrae. The left L4 spinal nerve was exposed carefully by blunt dissection and removal of the overlying transverse vertebral process, followed by tight ligation using the 6–0 silk suture and transection at the distal ligation site under a surgical microscope. Identical procedures were carried out in sham groups with an exception for ligation and transection.

### Behavioral Tests

All mice were habituated 1 to 2 h every day for 2 to 3 days before basal behavioral testing. Behavioral testing was accomplished by one investigator who was blinded with the group. Five behavioral tests were carried out as described previously, including mechanical, thermal, and cold tests, in addition to locomotor activity and conditional place preference (CPP) tests [25–28]. Each evoked behavioral test was committed at 30-min intervals.

Paw withdrawal thresholds in response to mechanical stimuli were measured with two calibrated von Frey filaments (0.07 and 0.4 g, Stoelting Co.). Briefly, the animals were placed inside individual Plexiglas chambers onto the rising mesh screen and allowed to habituate for 30 min. Each von Frey filament was applied to the plantar sides of both the hind paws ten times at the intervals of 5 min. The rapid paw withdrawal was regarded as a positive response. The number of positive responses among ten tests was recorded as percentage paw withdrawal frequency (% response frequency = (paw withdrawal number/10 test) × 100%).

Paw withdrawal latencies in response to noxious heat stimuli were examined with a Model 336 Analgesia Meter lightbox (IITC Inc. Life Science Instruments. Woodland Hills, CA). Briefly, the animals were put into individual Plexiglas chambers onto the surface of the glass plate. A beam of light was emitted from a hole in the lightbox and applied to the middle of the hind paw plantar surface. An automatic light beam shut was completed in the case of foot withdrawal by the mouse. Then, the duration from light beam starts to shut was recorded as paw withdrawal latency. We conducted this test 5 times for each side of every animal at intervals of 5 min. A cutoff time of 20 s was applied to avoid tissue damage to the hind paw.

Paw withdrawal latencies to noxious cold (0 °C) were examined. The mice were also placed in the individual Plexiglas chambers under 0 °C onto a cold aluminum plate, the temperature of which was monitored continuously by a thermometer. Then, the period from placing the animal onto the plate to a quick jump with/without paw flinching/shanking on the ipsilateral side was recorded as paw withdrawal latency. This test was repeated three times for each animal at 10-min intervals. The threshold time was set at 20 s to avoid damage to surrounding tissues.

CPP test was carried out as described with minor modifications [25]. Briefly, the CPP device comprising two different Plexiglas chambers connected to the inside gate (MED Associates In., St. Albans, VT) was employed. One of the chambers was composed of black/white horizontal stripes wall and a rough floor, while the other had black/white vertical stripes and a smooth floor. The photo beam detector was installed against the top of chamber walls to monitor mouse motion, and software was utilized to record time spent in every chamber automatically. Firstly, the mice could enter the two chambers for 30 min. After this pre-conditioning, the baseline time spent within every chamber in a 15-min period was recorded. For subsequent testing, the mice spending lower than 180 s or over 720 s in either chamber were eliminated. The inside gate was closed within the next 3 days, and the conditioning protocols were executed. In the morning on day 1, the mice were pooled with intrathecally injected 5 µL saline in one conditioning chamber. After 6 h in the afternoon, the mice were pooled with intrathecal injection of 0.8% lidocaine (dilution in 5 µL of saline) in the other conditioning chamber. The lidocaine and saline injection procedures were changed daily. On day 4 (examination day), the mice were allowed to enter the two chambers. The duration spent in an individual chamber within 15 min was recorded. The difference score was the difference between the basal and test durations spent within the lidocaine chamber.

Prior to euthanasia, locomotor activity tests were conducted, including placing, righting, and grasping reflexes. Placing reflex was measured when the dorsal surface of the hind-paws came in contact with the table edge, with the hind-limb position slightly inferior to forelimbs. The investigator recorded the reflexive or non-reflexive placement of hind-paws onto the table surface. Righting reflex was performed with the mouse back onto the flat surface. The investigator recorded the immediate returning of mice to the normal upright position. A grasping reflex was performed with the animals being placed onto the wire grid. The investigator recorded the grasping of wire-on-contact by the hind-paws. All the trials are repeated five times with a 10 min intermission. The scores of each reflex were determined based on counts of each normal reflex.

### Dorsal Root Ganglion Microinjection

DRG microinjection was conducted according to the previous description after slight modification [29, 30]. In brief, an incision was made in the middle of the lower lumbar back region after the mouse was anesthetized with sevoflurane. Articular processes in L4 or L3/L4 vertebrae were further dissected with caution and eliminated using small rongeurs. Following DRG exposure, the siRNA (1 µL, 20 mM) or viral (1 µL, 2 × 10^14^ TU/mL) solution were injected in unilateral L4 DRG or L3/L4 DRGs using a Hamilton syringe connected to a glass micropipette. After each DRG injection, the glass micropipette was retained for 10 min before removing it. Sterile saline was used to inundate the surgical field, followed by surgical incision closure using metal wound clips. Animals that exhibited aberrant locomotor activities were eliminated.

### Cell Culture and Transfection

According to previously described methods, DRG neurons and HEK-293 T cells were cultured [31, 32]. Briefly, HEK-293 T cells were cultivated in the DMEM/high glucose (HG) medium (Gibco ThermoFisher Scientific, Waltham, MA, USA) that contained 1% antibiotics and 10% fetal bovine serum (FBS) (Gibco ThermoFisher Scientific, Waltham, MA, USA). To prepare the primary DRG neuron culture, 4% sevoflurane was first used to euthanize CD1 male mice (4-week-old), then bilateral DRGs were collected in a cold neurobasal medium (Gibco Thermo Fisher Scientific, Waltham, MA, USA) that contained 10% FBS and 1% antibiotics. The tissues were incubated with Hanks’ balanced salt solution (HBSS) (Gibco Thermo Fisher Scientific, Waltham, MA, USA), including 1 mg/mL collagenase type I and 5 mg/mL disposed of for a 2-h period at 37 °C, followed by 10 min of digestion using 0.25% trypsin (Cellgro) at 37 °C and then using 0.25% trypsin inhibitor (Sigma). After trituration and centrifugation, the dissociated neurons were resuspended in a neurobasal defined medium containing 2% B27 supplement (Invitrogen), which were later placed into the 6-well plate containing poly-D-lysine (50 µg/mL, Sigma). The neurons were further cultivated at 37 °C and in 5% CO_2_. After 24 h, 2 µL of AAV5 (titer ≥ 1 × 10^13^/µL) or 100 nM siRNA (diluted with Lipofectamine 2000) was supplemented to 2-mL each well. After 3 days, the culture neurons were harvested for Western blot.

### WB Analysis

Four unilateral L4 DRGs collected in 4 animals were mixed to prepare the homogenate to achieve adequate protein for analysis. The tissues were homogenized and the cultured cells ultrasonicated in chilled lysis buffer (10 × 10^–3^ M Tris, 1 × 10^–3^ M phenylmethylsulphonyl fluoride, 5 × 10^–3^ M MgCl_2_, 5 × 10^–3^ M EGTA, 1 × 10^–3^ M EDTA, 1 × 10^–3^ M DTT, 40 × 10^–6^ m leupeptin, 250 × 10^–3^ M sucrose). The homogenates were centrifuged at 4 °C for 10 min at 10,000 g. Later, supernatants were obtained to analyze the cytosolic proteins and the centrifuged pellets to analyze the nuclear proteins. This study utilized the BCA protein assay kit (Pierce, Rockford, USA) to determine protein content. After heating for 6 min at 95 °C, equivalent quantities of protein (30 µg/Lane) were loaded onto a 4–15% stacking/7.5% resolving SDS-PAGE gel (Bio-Rad Laboratories) followed by electrophoretically transferred onto the PVDF membranes (250 mA, 2 h). The membranes were first blocked for 1 h with 5% skimmed milk in Tris-buffered supplemented with 0.1% Tween-20 at 37 °C. Later, primary antibodies were used to incubate membranes overnight at 4 °C, including against rabbit anti-C/EBPβ; (1:1000, ab32358, Abcam, USA), rabbit anti-TLR7 (1:500; ab113524, Abcam, USA), rabbit anti-GAPDH (1:2000; cat88845, CST, USA), rabbit anti-H3 (1:1000; PA5–16,183, Invitrogen, USA), mouse anti-GFAP (1:1000; ab7260, Abcam, USA), rabbit anti-phosphorylated ERK (p-ERK; 1:1000; ab207470, Abcam, USA), and rabbit anti-total ERK (1:1000; ab109282, Abcam, USA). Membranes were further incubated with HRP-labeled anti-rabbit or anti-mouse secondary antibody (1:2000; Invitrogen, USA) and visualized using RapidStep™ ECL Reagent (345,818, Merck Millipore, USA) and exposure using ChemiDoc Systems with ImagePro Lab software (Bio-Rad, USA). ImagePro software was used to capture images and perform densitometric analysis on the bands. To normalize against all cytosol proteins, GAPDH was used, whereas histone H3 was used to normalize against all nucleus proteins.

### qRT-PCR Assay

Total RNAs were extracted from DRGs using the RNA-Solv Reagent (Omega, BioTeK, GA) following the manufacturer’s instructions. Reverse transcription was organized with 2 µg of RNA using ReverTra Ace (TOYOBO, Osaka, Japan) and Oligo(dT) (TaKaRa, Japan). Then, the prepared cDNA was run in the 20-µL reaction system containing Advanced Universal SYBR Green Supermix (10 µL, Bio-Rad Laboratories, USA) and forward and reverse primers (Table 1; 250 nM each) in triplicate. The ViiA 7 Real-Time PCR System (Thermo Fisher Scientific, Waltham, USA) was employed to carry out qRT-PCR procedures. The cycling parameters were as follows: the mixture was incubated at 95 °C for 5 min, followed by 45 cycles at 95 °C for 30 s, 62 °C for 30 s, and 72 °C for 10 s. The relative mRNA transcript level was determined by the ΔΔCt method.

**Table 1:** Primers used

Names	Sequences
Single-cell RT-PCR	
*Tlr7* F	5′-GGTATGCCGCCAAATCTAAA-3′
*Tlr7* R	5′-GCTGAGGTCCAAAATTTCCA-3′
*Cebp-β* F	5′-CAAGCTGAGCGACGAGTACA-3′
*Cebp-β* R	5′-CAGCTGCTCCACCTTCTTCT-3′
*Gapdh* F	5′-TCGGTGTGAACGGATTTGGC-3′
*Gapdh* R	5′-TCCCATTCTCGGCCTTGACT-3′
ChIP RT-PCR	
*Tlr7*-promoter F	5′-AGGACAGGTTGCTTTATCAGGT-3′
*Tlr7*-promoter R	5′-TAACTTACACCACACGGGGG-3′
Luciferase	
*Tlr7* F	5′-CGGGGTACCAACCTAAACCACACAGCCCC-3′
*Tlr7* R	5′-CCCAAGCTTACAGAAAACCGAGACTCGCA-3′

*RT* reverse-transcription, *F* forward, *R* reverse

Our study conducted single-cell RT-PCR assays according to the previous description [33, 34]. In brief, the fresh DRG neurons from 3 to 4 weeks old mice were prepared. Three hours after plating, the size of the living single-DRG-neuron was calculated using an inverted microscope, and neurons were harvested using the glass microneedle. The collected neurons were placed in individual PCR tubes using cell lysis buffer (10 µL, Thermo Fisher Scientific). The cells were incubated for 10 min on ice and centrifuged at 4 °C and 10,000 g for 5 min to collect the supernatants. RT-PCR was conducted according to specific protocols using the single-cell RT-PCR assay kit (Thermo Fisher Scientific, Waltham, USA). The primers for single-cell RT-PCR are listed in Table 1.

### Plasmid Construction and Virus Production

The pcDNA3.1( −) plasmid harboring full-length mouse C/EBPβ was offered by Dr. Xi Li (Fudan University, Shanghai, China) [35]. After XbaI/BamHI digested the plasmid, C/EBPβ cDNA was purified and ligated into pro-viral plasmids (UNC Vector Core, CA). The C/EBPβ-expression vector was obtained, controlled by the cytomegalovirus promoter. DNA sequencing was performed to verify the recombinant clones. Our study adopted the AAV-DJ Helper Free Packaging System (Cell Biolabs, Inc., CA) to prepare AAV-DJ viral particles and purified using the AAV pro Purification Kit (Takara, Mountain View, CA).

### ChIP Assay

ChIP assays were performed based on the EZ-ChIP kit (Millipore, Germany), following the manufacturer’s instructions with minor modifications. Five unilateral L4 DRG from 5 SNL or sham mice were harvested and put together. After the homogenization, 1% formaldehyde was adopted for 10 min of homogenate cross-linking at 37 °C; then, 0.125 M glycine solution was added to quench reaction under ambient for 5 min. Further, the pellet was obtained by cold centrifugation at 1000 rpm for 5 min. After washing, the obtained pellet was prepared into suspension with protease inhibitor cocktail-containing lysis buffer that included 0.1% SDS, 1% NP-40, and 1% sodium deoxycholate. The chromatin was sheared by sonication of the suspension to generate the DNA fragments (0.2–1 kb). Subsequently, protein G agarose was added to pre-clear the samples under 4 °C for 2 h and immunoprecipitated using normal rabbit serum (2 µg) or rabbit anti-C/EBPβ antibody (2 µg, Abcam, USA) at 4 °C overnight, with 10% sample being the positive control for immunoprecipitation. PCR or qRT-PCR assays were conducted to purify and identify the DNA fragments. Table 1 presents all the primers used.

### Dual-Luciferase Assay

The 752-bp fragment (− 689 to + 62) of the *Tlr7* gene promoter region (including 2 C/EBPβ-binding motifs) was amplified based on the genomic DNA (gDNA) using PCR to manufacture *Tlr7* gene reporter plasmid. Table 1 lists the primers used. PCR products were inserted into the firefly luciferase reporter gene-containing pGL3-Basic vector by adopting Hind-III and Kpn-I restriction sites. DNA sequencing was performed later to confirm recombinant plasmid sequences.

HEK-293 T cells were cultured as described above. After incubation (24 h), Lipofectamine 2000 (Invitrogen, USA) was used to transfect the cells using URL-TK plasmid (40 ng, the Revilla luciferase reporter gene-containing control) with/without pGL3-Basic vectors (1 mg) in line with specific protocols. The cells were harvested in a passive lysis buffer 48 h after transfection. In triplicate, the luciferase activity was quantified in the supernatant using the Dual-Luciferase Reporter Assay System (Invitrogen, USA). The relative reporter activity was calculated when the firefly luciferase activity was normalized to refill activity.

### Statistical Analysis

Animals were randomly allocated to different treatment groups. The data were expressed as means ± SEM and analyzed by one-or two-way ANOVA (with a column factor treatment and row factor time’) and the paired, two-tailed Student’s *t*-test. The post hoc Tukey test was applied to compare means between two groups for statistically different results from ANOVA analysis. *P* < 0.05 was considered statistically significant. GraphPad Prism (GraphPad Software 8.0, USA) was used for the statistical analysis.

## Results

### C/EBPβ Expression Is Increased in Injured DRG after SNL

**Fig. 1:**
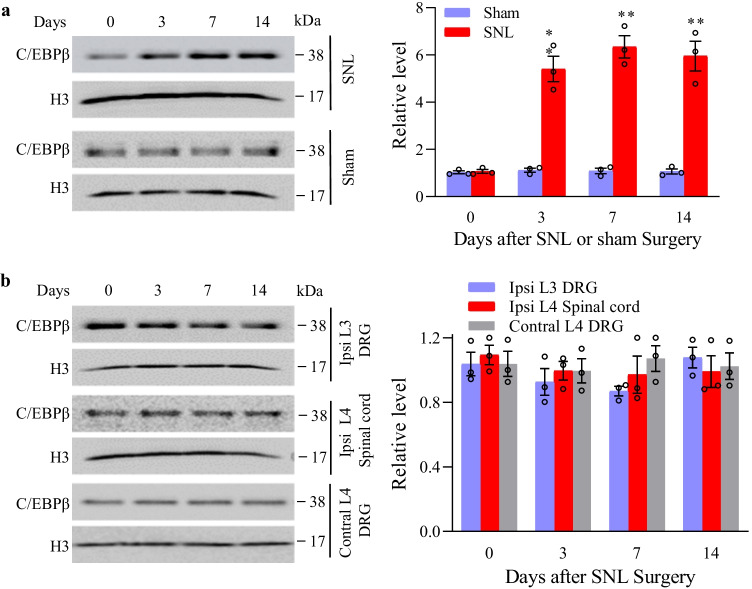
Peripheral nerve trauma-induced C/EBPβ protein increase in ipsilateral L4 DRG. **a** The protein level of C/EBPβ in ipsilateral L4 DRG in sham surgery or SNL mice. Typical Western blots (left panels) as well as densitometric analysis (right graphs). Three biological replicates were set (*n* = 12 mice) at every time point for every group. ^**^P < 0.01 vs. control (day 0) upon two-way ANOVA and post hoc Tukey test. **b** The protein level of C/EBPβ in ipsilateral L4 spinal cord, ipsilateral (intact) L3 DRG, and contralateral L4 DRG in SNL mice. Typical Western blots (left panels) as well as densitometric analysis (right graphs). Three biological replicates were set (*n* = 12 mice) at every time point. Two-way ANOVA and post hoc Tukey test

Before we explored the participation of C/EBPβ in SNL-induced TLR7 transcriptional activity in injured DRG, the change in C/EBPβ level was examined in two regions associated with pain, including DRG, and spinal cord, following SNL or sham surgery. Rather than sham surgery, SNL induced the constantly elevated C/EBPβ protein expression in the ipsilateral L4 (injured) DRG. On day 3 post-SNL, the expression of C/EBPβ protein increased 5.41–fold, while on days 7 and 14, it increased by 6.34- and 5.95-folds, respectively, compared with the matched value following sham surgery (Fig. 1a). Both SNL and sham surgery did not change the baseline C/EBPβ protein levels in ipsilateral L3 (uninjured) DRG, ipsilateral L4 spinal cord, and contralateral L4 DRG (Fig. 1b). SNL-induced C/EBPβ increase in DRG indicates an underlying role of C/EBPβ in neuropathic pain.

### Blocking the Increased C/EBPβ in Injured DRG Attenuates SNL-Induced Neuropathic Pain

**Fig. 2:**
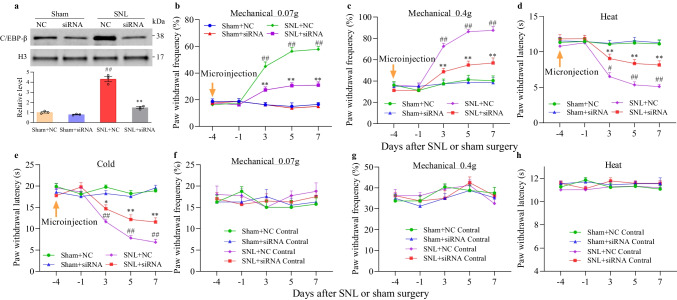
Blocking DRG C/EBPβ increase attenuates the occurrence of mouse neuropathic pain. siRNA: siRNA-C/EBPβ. NC, NC-siRNA. **a** The protein expression of C/EBPβ in ipsilateral L4 DRG on day 7 following sham surgery or SNL of treatment groups. Typical Western blots as well as densitometric analysis. Three biological replicates were set (*n* = 12 mice) for every group. One-way ANOVA and post hoc Tukey test. ^*##*^*P* < 0.01 versus the siRNA NC-microinjected sham mice. ^**^P < 0.01 versus the siRNA NC-microinjected SNL mice. **b–h** Effect of pre-microinjection of NC-siRNA or siRNA-C/EBPβ into the ipsilateral L4 DRG on paw withdrawal responses to 0.07 g von Frey filament (**b**, **f**), 0.4 g von Frey filament (**c**, **g**), thermal (**d, h**) and cold (**e**) stimuli on the ipsilateral (**b–e**) and contralateral (**f–h**) sides in mice as indicated days after sham surgery or SNL. *n* = 12 mice in each group. Two-way ANOVA and repeated measurements as well as post hoc Tukey test. ^#^*P* < 0.05 and ^##^*P* < 0.01 vs. the siRNA-negative control microinjected sham mice at the corresponding time points. ^**^P < 0.01 versus siRNA NC-microinjected SNL mice at the corresponding time point

In order to study whether C/EBPβ upregulation in injured DRG involved neuropathic pain generation, we examined whether blocking C/EBPβ increase in the injured DRG affected SNL-mediated pain hypersensitivity induction. To this end, specific C/EBPβ small interfering RNA (siRNA) was pre-microinjected into the ipsilateral L4 DRG 4 days prior to SNL or sham surgery. As expected, at 7 days post-SNL, the expression of C/EBPβ in ipsilateral L4 DRG elevated in animals with microinjection of negative control siRNA (NC-siRNA), compared with sham surgery group administered with microinjection of negative control siRNA (Fig. 2a). SNL mice with C/EBPβ siRNA pre-microinjection did not show elevated C/EBPβ expression (Fig. 2a). There was no significant alteration in the basal C/EBPβ protein expression in ipsilateral L4 DRG from mice given microinjection of C/EBPβ siRNA (Fig. 2a). Based on these findings, pre-microinjection with C/EBPβ siRNA prevented the SNL-mediated C/EBPβ upregulation in injured DRG.

Consistent with previous studies [22, 23, 26], SNL produced permanent ipsilateral cold/thermal hyperalgesia and mechanical allodynia in mice given microinjection of NC-siRNA (Fig. 2b–e). Relative to baseline data before injection, at 3, 5, and 7 days after SNL, the paw withdrawal frequencies of ipsilateral hind-paw in response to mechanical stimuli elevated significantly (Fig. 2b, c). Besides, for ipsilateral hind-paw, its paw withdrawal latency upon cold and thermal stimuli decreased dramatically at 3, 5, and 7 days after SNL compared to before injection (Fig. 2d, e). In addition, C/EBPβ siRNA pre-microinjection had no difference to ipsilateral paw responses in the presence of thermal/cold/mechanical stimuli in mice receiving sham surgery (Fig. 2b–e) and reduced the SNL-mediated cold/thermal hyperalgesia and mechanical allodynia (Fig. 2b–e). Expectedly, SNL-mediated cold/thermal hyperalgesia and mechanical allodynia were still observed in the ipsilateral side of negative control siRNA-microinjected mice in the observation process (Fig. 2b–e). Besides, siRNA pre-microinjection had no difference to contralateral baseline paw responses of SNL and sham mice (Fig. 2f–h) or the locomotor activity (Table 2).

**Table 2:** Locomotor tests

Treatment groups	Locomotor function tests		
	Placing	Grasping	Righting
NC siRNA + Sham	5 (0)	5 (0)	5 (0)
Cebp-β siRNA + Sham	5 (0)	5 (0)	5 (0)
NC siRNA + SNL	5 (0)	5 (0)	5 (0)
Cebp-β siRNA + SNL	5 (0)	5 (0)	5 (0)
AAV5-GFP	5 (0)	5 (0)	5 (0)
AAV5-C/EBP-β	5 (0)	5 (0)	5 (0)

Values are means (SEM). *n* = 10–12 mice/group; 5 trials

*NC* negative control

**Fig. 3:**
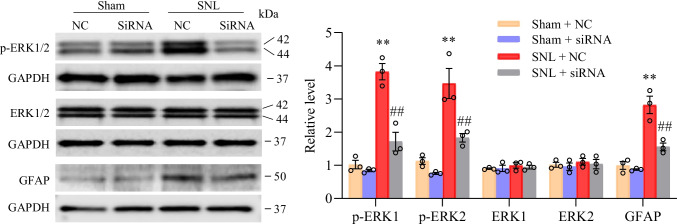
Role of siRNA-C/EBPβ pre-microinjection to ipsilateral L4 DRG in the central sensitization of dorsal horn, demonstrated by the SNL-mediated upregulation of ERK1/2 phosphorylation (p-ERK1/2) together with GFAP expression in ipsilateral L4 dorsal horn at 7 days following sham surgery or SNL. Typical Western blots (left panels) together with densitometric analysis (right graphs) are displayed. *n* = 3 biological repeats (12 mice) per group. One-way analysis variance (ANOVA) and post hoc Tukey test. ^**^P < 0.01 vs. relevant sham mice receiving NC-siRNA microinjection. ^##^*P* < 0.01 versus the corresponding siRNA NC microinjected SNL mice

This study further analyzed the influence of C/EBPβ siRNA pre-microinjection on the SNL-mediated central sensitization of dorsal horn and revealed by the increased phosphorylation of glial fibrillary acidic protein (GFAP, the astrocyte hyperactivation marker) and extracellular signal-regulated kinase 1/2 (p-ERK1/2, the neuronal hyperactivation marker) in the dorsal horn [21, 32]. Conforming to research based on neuropathic pain model induced by chronic constriction injury or spinal nerve injury [21, 32], at 7 days post-SNL, the protein expression of the phosphorylation of ERK1/2 (rather than total ERK1/2) and GFAP elevated significantly in ipsilateral L4 dorsal horn of SNL mice given microinjection of NC-siRNA, compared to the negative control siRNA-microinjected sham mice (Fig. 3). Such changes disappeared in SNL mice with microinjection of C/EBPβ siRNA (Fig. 3). Neither C/EBPβ siRNA nor negative control siRNA changed the baseline phosphorylation levels of GFAP, total ERK1/2, and ERK1/2 in the dorsal horn of mice receiving sham surgery (Fig. 3). Overall, based on this study, C/EBPβ upregulation in the injured DRG played an essential role in the central sensitization of the dorsal horn and pain hypersensitivity induced by SNL.

### Mimicking the SNL-Induced Increase in C/EBPβ in Injured DRG Leads to Neuropathic Pain Symptoms

**Fig. 4:**
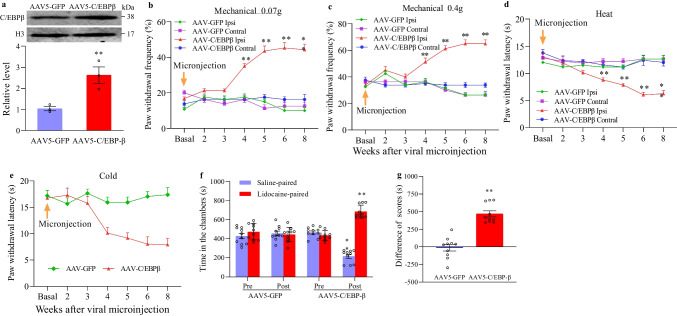
Overexpression of DRG C/EBPβ leads to symptoms of neuropathic pain. **a** The protein expression of C/EBPβ in ipsilateral L3/4 DRGs in week 8 post-control AAV5-GFP or AAV5-C/EBPβ microinjection. Three biological replicates were set (*n* = 6 mice) for each group. ^**^P < 0.01 vs. AAV5-GFP group upon unpaired two-tailed Student’s *t*-test. **b**–**e** Effect of DRG microinjection of AAV5-C/EBPβ or AAV5-GFP on paw withdrawal responses to 0.07 g von Frey filament (**b**), 0.4 g von Frey filament (**c**), thermal (**d**), and cold (**e**) stimuli on the ipsilateral (Ipsi) and contralateral (Contral) sides on weeks as indicated after viral microinjection into the unilateral L3/4 DRGs. *n* = 10 mice for each group. Two-way ANOVA and post hoc Tukey test versus the corresponding GFP group. BL: baseline. ^**^P < 0.01 versus ipsilateral control AAV5-GFP group at a specific time. **f**, **g** Effect of DRG microinjection of AAV5-GFP or AAV5-C/EBPβ into unilateral L3/4 DRGs on the spontaneous pain evaluated using the CPP paradigm. Post, post-conditioning. Pre, pre-conditioning. *n* = 10 mice for each group. Two-way ANOVA and post hoc Tukey test (**f**) or the independent, two-tailed Student’s *t*-test (**g**). ^**^P < 0.01 versus the corresponding pre-conditioning

This study also investigated the sufficiency of C/EBPβ upregulation in DRG for neuropathic pain mediated by SNL. In this regard, AAV5 expressing full-length C/EBPβ (AAV5-C/EBPβ) was microinjected in unilateral L3/L4 DRGs into naïve male adult mice, while AAV5 that expressed green fluorescent protein (AAV5-GFP) served as the control. C/EBPβ protein expression substantially increased in microinjected DRGs in week 8 following AAV5-C/EBPβ microinjection, rather than AAV5–GFP (Fig. 4a). Upon mechanical stimulation, mice given microinjection of AAV5-C/EBPβ (rather than AAV5–GFP) showed significantly increased paw withdrawal frequency (Fig. 4b, c) along with ipsilateral cold allodynia and thermal hyperalgesia, indicated by the decreased paw withdrawal latency upon cold and thermal stimuli, respectively (Fig. 4d, e). The nociceptive hypersensitivity appeared in the 4th week, which lasted for eight or more weeks (Fig. 4b–e), conforming to the 3–4-week lag period of AAV5 level that persisted for three or more months [21, 32]. AAV5-GFP and AAV5-C/EBPβ did not change the contralateral baseline paw responses upon cold/thermal/mechanical stimuli (Fig. 4b–e) or the locomotor activity (Table 2).

Besides, to test the stimuli-induced nociceptive hypersensitivity, the CPP paradigm was adopted to test the effect of mimicking SNL-mediated C/EBPβ upregulation in DRG on activating the spontaneous persistent nociceptive responses. The mice microinjected with AAV5-C/EBPβ exhibited a significant preference towards the lidocaine-paired chamber (Fig. 4f, g), demonstrating that the spontaneous pain response was independent of stimuli. The mice microinjected with AAV5-GFP showed no distinct preference for lidocaine-or saline-paired chamber (Fig. 4f, g), indicating no marked spontaneous pain responses. These data suggest that increased C/EBPβ in DRG produces typical neuropathic pain symptoms, as both spontaneous and evoked nociceptive hypersensitivity.

**Fig. 5:**
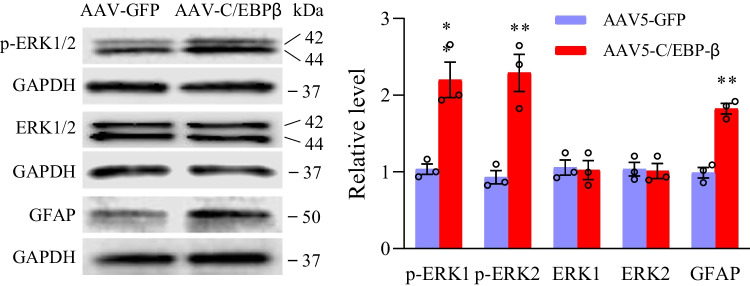
Effect of DRG overexpression of C/EBPβ achieved by AAV5-C/EBPβ microinjection into unilateral L3/4 DRGs on central sensitization in ipsilateral L3/4 dorsal horn, indicated by the increased ERK1/2 phosphorylation (p-ERK1/2) as well as GFAP expression in week 8 following microinjection. Typical Western blots (left panels) as well as densitometric analysis (right graphs). Independent, two-tailed Student’s *t*-test. Three biological replicates were set (*n* = 6 mice) for each group. ^**^P < 0.01 vs. related AAV5-GFP group

The above results obtained from behavioral tests were evidenced by central sensitization of the spinal cord dorsal horn. In week 8 post-AAV5-C/EBPβ microinjection, the GFAP and p-ERK1/2 levels elevated remarkably in the ipsilateral L3/4 spinal cord dorsal horn in comparison with post-AAV5-GFP microinjection (Fig. 5).

### C/EBPβ Transcriptionally Activates the Tlr7 Gene after SNL in Injured DRG

**Fig. 6:**
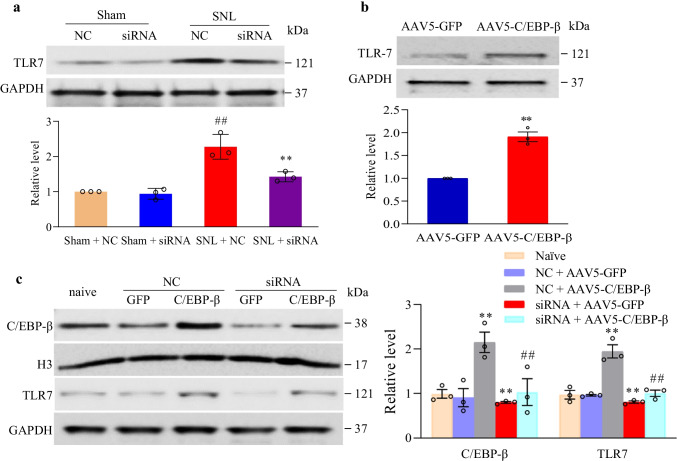
Participation of DRG increased C/EBPβ in nerve trauma-mediated TLR7 upregulation in injured DRG. **a** TLR7 expression in ipsilateral L4 DRG in mice microinjected with NC-siRNA or C/EBPβ siRNA on day 7 following sham surgery or SNL. Typical Western blots (left panels) as well as densitometric analysis (right graphs). Three biological replicates were set (*n* = 12 mice for each group). ^*##*^*P* < 0.01 vs. sham surgery group given NC-siRNA microinjection. ^**^P < 0.01 vs. SNL group given NC-siRNA microinjection upon one-way ANOVA and post hoc Tukey test. **b** TLR7 expression in L3/4 DRGs in week 8 following control AAV5-GFP or AAV5-C/EBPβ microinjection. *n* = 3 biological replicates (*n* = 6 mice for each group). ^**^P < 0.01 vs. AAV5-GFP group upon independent, two-tailed Student’s *t*-test. **c** TLR7 protein expression in cultured mouse DRG neurons. Three biological replicates were set for each treatment. One-way analysis variance (ANOVA) and post hoc Tukey test. ^**^P < 0.01 vs. related naïve group. ^*##*^*P* < 0.01 vs. related AAV5-C/EBPβ plus C/EBPβ siRNA-treated group

Our study also examined the role of SNL-mediated C/EBPβ upregulation in activating the *Tlr7* gene transcriptionally in injured DRG. At 7 days post-SNL, pre-microinjection of C/EBPβ siRNA in DRGs, rather than NC-siRNA, suppressed the SNL-mediated TLR7 protein increase in ipsilateral L4 DRG (Fig. 6a). At 7 days after sham surgery, pre-microinjection of C/EBPβ siRNA did not involve the basal TLR7 protein expression in ipsilateral L4 DRG (Fig. 6a). Consistently, in week 8, AAV5-C/EBPβ microinjection, rather than AAV5-GFP, in unilateral L3/4 DRGs led to TLR7 upregulation in microinjected DRG (Fig. 6b). For a better understanding of how C/EBPβ affected TLR7 level in DRG neurons, full-length C/EBPβ through AAV5-C/EBPβ was transduced into cultured DRG neurons to overexpress C/EBPβ. C/EBPβ overexpression significantly increased the TLR7 level (Fig. 6c). Such elevation disappeared in the cultured neurons after co-transfection with C/EBPβ*-*specific siRNA and AAV5-C/EBPβ (rather than NC-siRNA) (Fig. 6c), revealing TLR7 upregulation was specific in response to C/EBPβ. In addition, transfection of C/EBPβ siRNA alone also reduced the basal TLR7 expression (Fig. 6c). Thus, our data imply that C/EBPβ may directly regulate TLR7 in DRG neurons under neuropathic pain conditions.

**Fig. 7:**
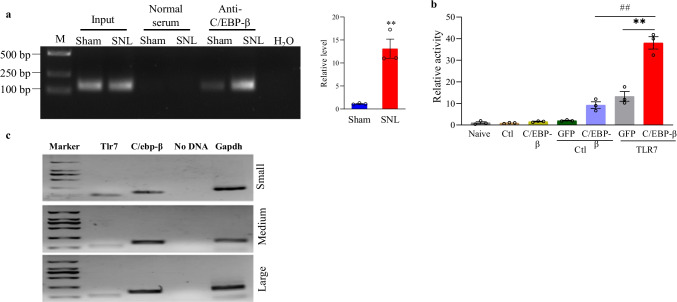
C/EBPβ binds to and triggers the *Tlr7* promoter in ipsilateral L4 DRG after injury to the peripheral nerve. **a** On the 7th day after sham surgery or SNL, a fragment in the *Tlr7* promoter was immunoprecipitated with the rabbit anti-C/EBPβ antibody in ipsilateral L4 DRG. M: ladder marker. Input: the whole *Tlr7* mRNA fragment purified. Three biological replicates (*n* = 30 mice) in each group. ^**^P < 0.01 upon unpaired two-tailed Student’s *t*-test compared with the sham group. **b** The activity of *Tlr7* promoter in vectors-and siRNAs-transfected HEK-293 T cells. Ctl, control vector (pGL3-basic). Naive, untreated. GFP, *Gfp*-expressing pro-viral vector. C/EBP-β, full-length C/EBPβ-expressing pro-viral vector. TLR7, *Tlr7* promoter-containing pGL3 reporter vector. *n* = 3 replicates for each treatment. ^**^P < 0.*01* compared with pGL3 *Tlr7* vector (*Tlr7*) alone upon one-way ANOVA and post hoc Tukey test. **c** C/EBPβ mRNA was co-expressed with *Tlr7* mRNA in lumbar DRG neurons of all sizes (small, moderate, large). Marker: ladder marker. *n* = 3 biological replicates

To better confirm whether C/EBPβ directly affected *Tlr7* gene expression, ChIP assays were carried out. At 7 days after sham surgery, a *Tlr7* promoter fragment, including C/EBPβ binding motifs, was amplified from C/EBPβ antibody-immunoprecipitated complex from ipsilateral L4 DRGs in nuclear fractions (Fig. 7a). However, amplification using normal serum could not be conducted (Fig. 7a), indicating that the binding of C/EBPβ to *Tlr7* promoter was specific. At 7 days post-SNL, C/EBPβ had markedly elevated binding activity to the *Tlr7* promoter in injured DRG, evidenced by the 13 times increased band density in ipsilateral L4 DRGs of SNL mice in comparison with sham mice (Fig. 7a). Based on these results, binding at 7 days post-SNL was possibly ascribed to the elevated C/EBPβ protein expression in ipsilateral L4 DRG (Fig. 1a). Moreover, co-transfection with the full-length C/EBPβ vector, rather than the control *Gfp* vector, evidently enhanced the *Tlr7* gene promoter’s transcription activity, as evidenced by luciferase assay results using HEK-293 T cells (Fig. 7b). Additionally, C/EBPβ mRNA was co-expressed with TLR7 mRNA in all DRG neurons of all sizes (small, moderate, large), as evidenced by single-cell RT-PCR assay (Fig. 7c). These data suggest that the SNL-mediated C/EBPβ upregulation promotes the *Tlr7* promoter binding activity, leading to the increases in TLR7 mRNA transcription and translation in injured DRG.

## Discussion

Peripheral neuropathic pain primarily resulted from peripheral nerve injury leading to DRG neurons with axotomized (non-conducting) and uninterrupted fibers, which may conduct in the corresponding receptive fields. In human beings, neuropathic pain has the feature of spontaneous pain or evoked pain caused by hypersensitivity to the normally non-painful (allodynia) or painful (hyperalgesia) stimuli. Investigating how peripheral nerve trauma produces hypersensitivity may provide a new route to understand, prevent, and treat this disturbance. Our previous study showed that SNL induced upregulated expression of TLR7 in the injured DRG [10]. The present study further exhibited that this upregulation was attributed to the peripheral nerve trauma-induced increase in C/EBPβ in injured DRG. Based on our findings, C/EBPβ was involved in transcriptionally activating the *Tlr7* gene in injured DRG neurons under neuropathic pain conditions.

C/EBPβ belongs to the C/EBPs family with six members that have been reported, which are basic-leucine zipper transcription factors that interact with CCAAT motifs present in numerous gene promoters. Similar to TFs like myeloid zinc finger [28, 29], octamer transcription factor 1 [24, 36], and runt-related transcription factor 1 [21], C/EBPβ expression can be regulated following peripheral nerve trauma. A previous study revealed that CCI upregulated C/EBPβ expression at protein and mRNA levels in the ipsilateral L3/4 DRGs [17]. However, whether C/EBPβ expression in intact/adjoining DRG was altered following peripheral nerve trauma is unclear. The present study demonstrated that SNL persistently enhanced the C/EBPβ protein level in the ipsilateral L4 DRG, rather than the intact/adjacent L3 DRG, ipsilateral L4 spinal cord, and contralateral L4 DRG, during the observation period. Based on these results, such increase only occurs in injured DRG neurons, as C/EBPβ mRNA was identified in small, medium, and large DRG neurons, rather than satellite glial cells, of naive mice [17]. In addition, in the CCI mouse model, in the ipsilateral L3/4 DRGs, many neurons labeled with C/EBPβ mRNA were positive for ATF3, an injury marker. Interestingly, the level of C/EBPβ in injured DRG on day 14 post-CCI was significantly higher than the control group but markedly decreased as compared to on day 7 post-CCI [17]. This indicates that the temporal pattern of C/EBPβ expression in injured DRG is incompletely the same between SNL and CCI models. How peripheral nerve trauma results in the increased DRG C/EBPβ mRNA expression remains unclear, and this upregulation may be associated with other TFs or enhanced RNA stability or epigenetic modifications, which should be further explored in the future.

C/EBPβ participates in peripheral nerve trauma-induced transcriptional activation of *Tlr7* promoter in injured DRG. TLRs play critical roles in stimulating innate immune responses against molecular patterns related to pathogens in mammals. TLR7 as a member of the TLRs is an endosomal innate immune sensor to recognize single-stranded RNAs [9]. In DRG neurons, TLR7 was positive for MrgprA3, CGRP, TRPA1, and TRPV1 [10–12], indicating that TLR7 may serve as an innovative pain mediator in nociceptive neurons. Indeed, we previously demonstrated that TLR7 protein and mRNA expression increased in injured DRG neurons in the SNL mouse model [10]. Suppressing such increased expression mitigated the SNL-mediated nociceptive hypersensitivity in male and female mice’s growth and maintenance [10]. Mimicking this upregulation strengthened the responses to cold/thermal/mechanical stimuli [10]. Mechanistically, the upregulated TLR7 triggered the nuclear factor kappa B (NF-κB) pathway by increasing nuclear import and phosphorylation of p65 in injured DRG neurons [10]. Thus, DRG upregulated TLR7 promotes neuropathic pain, possibly by the activation of NF-κB in the nociceptive primary sensory neurons. It is essential to understand how the *Tlr7* gene is transcriptionally activated in injured DRG post-SNL. The present study demonstrated that blocking DRG increased C/EBPβ by DRG microinjection of C/EBPβ siRNA attenuated SNL-mediated TLR7 upregulation in injured DRG. DRG overexpression of C/EBPβ elevated the expression of TLR7 in the injected DRG. These in vivo findings were further confirmed in vitro in DRG neuronal culture. To our surprise, C/EBPβ siRNA microinjection had no significant difference to the baseline TLR7 or C/EBPβ protein expression of the sham group, even though siRNA showed high efficiency in cultured DRG neurons. The underlying cause of the above phenomenon is uncertain but might be due to its lower level that could not be further reduced by C/EBPβ siRNA at the dosage used in DRG normally. Furthermore, C/EBPβ mRNA may have higher translational efficacy after knockdown, allowing the sham group to maintain normal baseline C/EBPβ protein expression in injured DRG. The present study also demonstrated C/EBPβ binding to the *Tlr7* promoter and increased this binding after SNL in injured DRG. Given that both *Cebpβ* mRNA and *Tlr7* mRNA co-expressed in small, medium, and large DRG neurons, C/EBPβ directly stimulated *Tlr7* promoter activation in the individual DRG neurons. Overall, the above results illustrate that C/EBPβ has a vital function in the peripheral nerve trauma-induced increase of TLR7 in injured DRG.

C/EBPβ promotes neuropathic pain’s genesis, likely through the triggered activation of *Tlr7* in injured DRG. The evidence from the present work and previous study [17] revealed that blocking DRG increased C/EBPβ and mitigated the development of nociceptive hypersensitivity caused by CCI or SNL. C/EBPβ overexpression in DRG produced spontaneous pain and increased responses to mechanical, thermal, and cold stimulation [17]. The evidence indicates the participation of DRG increased C/EBPβ in nerve trauma-induced nociceptive hypersensitivity. As discussed, SNL-induced upregulation of DRG TLR7 mediates this participation. However, the involvement of other downstream targets of C/EBPβ cannot be excluded. For example, the previous study showed C/EBPβ role in peripheral nerve trauma-induced upregulation of euchromatic histone-lysine *N*-methyltransferase 2 in injured DRG [17]. Multiple mechanisms may mediate the role of increased C/EBPβ in DRG in neuropathic pain genesis.

## Data Availability

The data that support the results of this study are available from the corresponding author upon reasonable request.

## Declarations

### Welfare of Animals

All procedures performed in studies involving animals were approved by the ethical standards of Institutional Animal Care and Use Committees of Zhengzhou University, Zhengzhou 450052, China. Mice received humane care following the Guidelines for the Care and Use of Laboratory Animals of the National Institutes of Health.
